# Supplementary material for: The Effect of Strain Level Diversity on Robust Inference of Virus-Induced Mortality of Phytoplankton
Source: Front Microbiol. 2018 Sep 3;9:1850. doi: 10.3389/fmicb.2018.01850 (PMC6129610; doi:10.3389/fmicb.2018.01850)
Supplement: Supplementary file 1 [file Data_Sheet_1.PDF]

Supplementary Material:
The effect of strain level diversity on robust inference of virus-induced mortality

1 SUPPLEMENTAL DATA A: STEADY STATES

Model 1

In the baseline model, equation 2, the steady state densities for phytoplankton (P), viruses (V) and grazers (G) are given as:

$$P^* = \frac{\omega}{(\beta - 1)\,\phi} \tag{S1}$$

$$V^* = \frac{r\left(1 - \frac{\omega}{(\beta-1)\phi K}\right) - aG_0}{\phi} \tag{S2}$$

$$G^* = G_0 \tag{S3}$$

Extension 1: infected class

In the infected class extension described by the system of equations given in equation 4, the steady state densities for susceptible phytoplankton, infected phytoplankton (I), viruses and grazers are given as:

$$P^* = \frac{\omega}{\phi\left(\frac{\beta\eta}{\eta+aG_0} - 1\right)} \tag{S4}$$

$$I^* = \left(\frac{\omega}{\eta\,(\beta - 1) - aG_0}\right) V^* \tag{S5}$$

$$V^* = \frac{r\left(1 - \frac{\omega(\eta+aG_0)}{K\phi(\eta(\beta-1)-aG_0)}\right) - aG_0}{\phi\left(1 + \frac{r\omega}{K\phi(\eta(\beta-1)-aG_0)}\right)} \tag{S6}$$

$$G^* = G_0 \tag{S7}$$

Extension 2: diversity

The steady state densities for the model described in equation 6 are given for the different phytoplankton strains, different virus strains and grazers as:

$$P_1^* = \frac{\omega_1}{(\beta_1 - 1)\,\phi_1} \tag{S8}$$

$$P_2^* = \frac{\omega_2}{(\beta_2 - 1)\,\phi_2} \tag{S9}$$

$$V_1^* = \frac{1}{\phi_1}\left(r_1\left(1 - \frac{1}{K}\left(\frac{\omega_1}{(\beta_1 - 1)\,\phi_1} + \frac{\omega_2}{(\beta_2 - 1)\,\phi_2}\right)\right) - a_1 G_0\right) \tag{S10}$$

$$V_2^* = \frac{1}{\phi_2}\left(r_2\left(1 - \frac{1}{K}\left(\frac{\omega_1}{(\beta_1 - 1)\,\phi_1} + \frac{\omega_2}{(\beta_2 - 1)\,\phi_2}\right)\right) - a_2 G_0\right) \tag{S11}$$

$$G^* = G_0 \tag{S12}$$

SUPPLEMENTAL DATA B: PARTITIONING MORTALITY IN THE BASELINE MODEL

Partitioning between top-down and bottom-up mortality at steady state – model 1

The condition for the steady state of the baseline model in equation 2 leads to a balance of gains and losses:

$$r\left(1 - \frac{\omega}{(\beta - 1)\,\phi K}\right) = aG_0 + \phi V^* \tag{S13}$$

which shows niche-competiton limited growth is equal to the top-down pressures of grazers and viruses. This makes it possible to change life history traits to set the relative bottom-up (niche competition) to top-down mortality, and partition top-down mortality between grazing and viral induced lysis.

For a relative amount of bottom-up mortality x then the following condition (niche competition : growth rate) must be satisfied:

$$x = \frac{\omega}{(\beta - 1)\,\phi K}. \tag{S14}$$

Rewriting, we can set K as:

$$K = \frac{\omega}{(\beta - 1)\,\phi x}. \tag{S15}$$

The relative top-down mortality is thus given by: $1 - x$. Within this fraction we can partition between grazing and lysis. For a proportion of grazing y:

$$y = \frac{aG_0}{r\left(1 - \frac{\omega}{(\beta-1)\phi K}\right)}. \tag{S16}$$

Then a can be set as:

$$a = \frac{yr\left(1 - \frac{\omega}{(\beta-1)\phi K}\right)}{G_0}. \tag{S17}$$

By first setting x and looking across values for y in [0,1] it is possible to search the niche competition isoclines indicated in Figure 2.

SUPPLEMENTAL DATA C: PARTITIONING MORTALITY WITH AN INFECTED CLASS

Partitioning between top-down and bottom-up mortality at steady state – model 2

The condition for the steady state of the infected class model in equation 4 leads to a balance of gains and losses for both infected and susceptible cells as:

$$rP^*\left(1-\frac{P^*+I^*}{K}\right)=a\left(P^*+I^*\right)G_0+\eta I^* \tag{S18}$$

Here, we find the relative botom-up mortality, x, as:

$$x=\frac{\frac{rP^*(P^*+I^*)}{K}}{rP^*}=\frac{P^*+I^*}{K} \tag{S19}$$

which gives:

$$K=\frac{P^*\left(\eta+r\left(1-x\right)\right)}{x\left(\eta+aG_0\right)}. \tag{S20}$$

Note that this formulation contains *all* model parameters. This means that we cannot independently set the exact amounts of niche competition, grazing and lysis simultaneously as done for the baseline model. However, we can still come up with an equation form to set the amount of lysis relative to grazing. The relative amount of lysis:grazing given by y can be calculated as:

$$y=\frac{\eta I^*}{a\left(P^*+I^*\right)G_0+\eta I^*} \tag{S21}$$

or, using equation S18:

$$y=\frac{\eta I^*}{rP^*\left(1-\frac{P^*+I^*}{K}\right)}. \tag{S22}$$

Using the latter, we find that:

$$K\left(1-\frac{aG_0\left(1+\frac{yr}{\eta}\right)}{r\left(1-y\right)}\right)=P^*. \tag{S23}$$

One can set x and y simulataneously by equating P^* in equation S23 with P^* in equation S20. Doing so, one finds a formulation for a in terms of x and y:

$$a=\frac{\eta\left(1-x\right)+r\left(1-x\right)}{G_0\left(x+\frac{\eta+yr}{r(1-y)}+\frac{\left(1+\frac{yr}{\eta}\right)(1-x)}{1-y}\right)}. \tag{S24}$$

The numerical implementation to simulate the dilution method first sets x and y, then calculates a using equation S24 and then calculates K using equation S20. In that way we can set the balance between niche comptition, grazing, and viral-induced lysis (see Figure 2).

SUPPLEMENTAL DATA D: ANALYTICAL EXPRESSIONS FOR DILUTION CURVE WITH INFECTED CLASS

The per capita growth rate of total phytoplankton in the infected class model described in equation 4 is found by summing the growth terms for both susceptible and infected phytoplankton and dividing by the total number of phytoplankton P+I:

$$\frac{\frac{dP}{dt}+\frac{dI}{dt}}{P+I}=\frac{1}{P+I}\left(rP\left(1-\frac{P+I}{K}\right)-a\left(P+I\right)G-\eta I\right). \tag{S25}$$

In doing so the terms for newly infected cells (lost from P and gained by I) cancel out. The instantaneous growth rates across dilution treatments are then found for the classic dilution series as:

$$\overbrace{\left.\frac{\frac{dP}{dt}+\frac{dI}{dt}}{P+I}\right|_{FP_0,FI_0,FG_0,V_0}}^{classic\,filter}=\overbrace{\left(\frac{rP_0-\eta I_0}{P_0+I_0}\right)}^{intercept}-\overbrace{\left(aG_0+\frac{rP_0}{K}\right)}^{slope}F \tag{S26}$$

and for the modified dilution series as:

$$\overbrace{\left.\frac{\frac{dP}{dt}+\frac{dI}{dt}}{P+I}\right|_{FP_0,FI_0,FG_0,FV_0}}^{modified\,filter}=\overbrace{\left(\frac{rP_0-\eta I_0}{P_0+I_0}\right)}^{intercept}-\overbrace{\left(aG_0+\frac{rP_0}{K}\right)}^{slope}F. \tag{S27}$$

As the state term for viruses does not appear in equation S25 the results are expected to be the same for both the classical and modified dilution curves. Hence, the instantaneous calculation of viral lysis, is predicted to be 0. We can also evaluate the virus dilution method, in which we find a similar expression for the same reason:

$$\overbrace{\left.\frac{\frac{dP}{dt}+\frac{dI}{dt}}{P+I}\right|_{P_0,I_0,G_0,FV_0}}^{virus-only\,filter}=\overbrace{\frac{rP_0-\eta I_0}{P_0+I_0}-\frac{rP_0}{K}-aG_0}^{intercept}. \tag{S28}$$

As viruses do not directly cause cell lysis in this model (rather it is indirect - via the infected class) we have not yet found easily interpretable analytical expressions for the dilution curve.

SUPPLEMENTAL DATA E: INFECTED CLASS MODEL BEHAVIOUR 2H

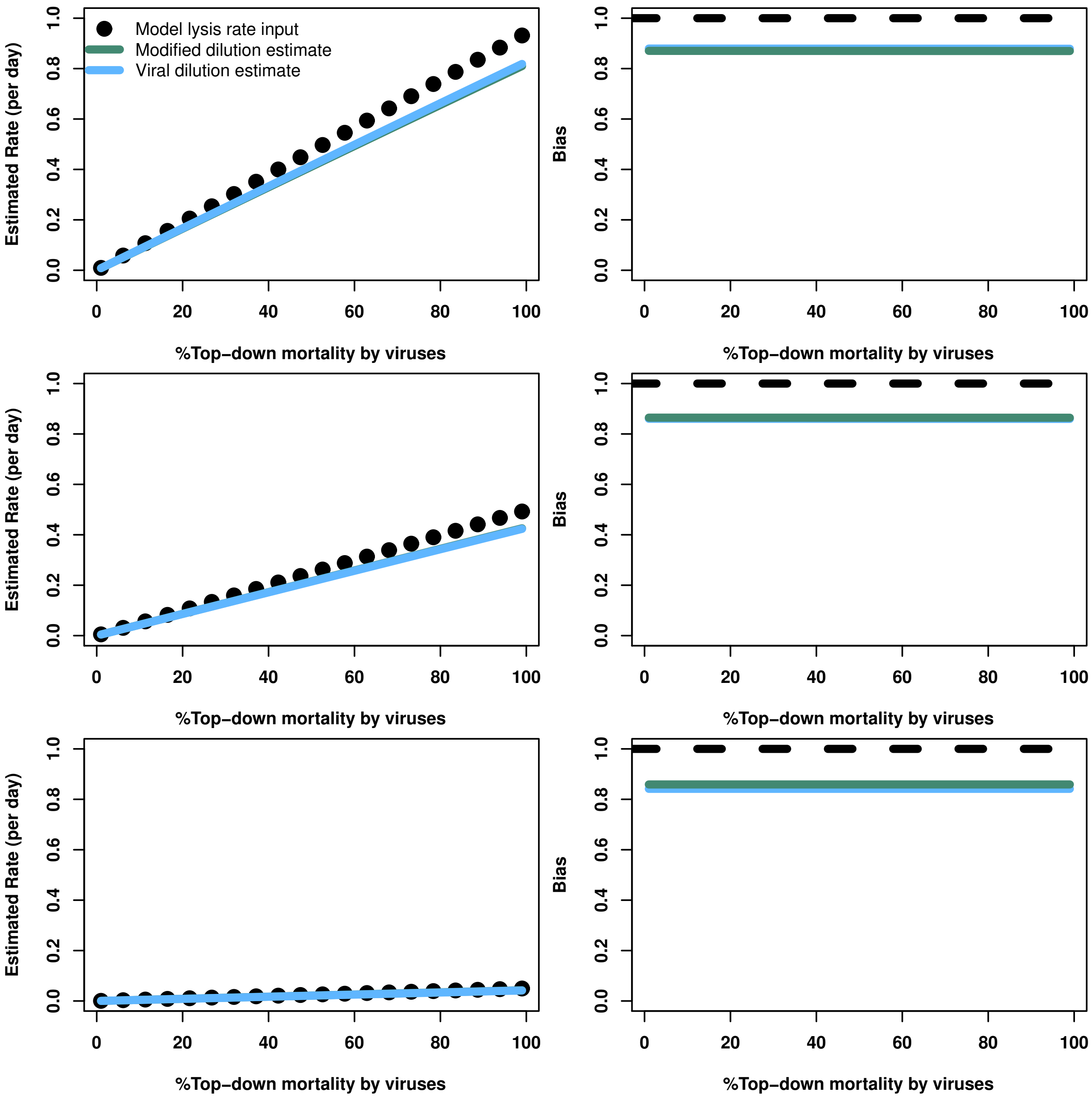

Figure S1. Rate estimates (left) and bias (right) of viral lysis rate in the infected class model with a 15 minute latent period calculated using a 2h incubation. Each row shows a different level of niche competition as indicated by the lines across Figure 2 (top: 5%, middle:50%, bottom: 95% niche competition).

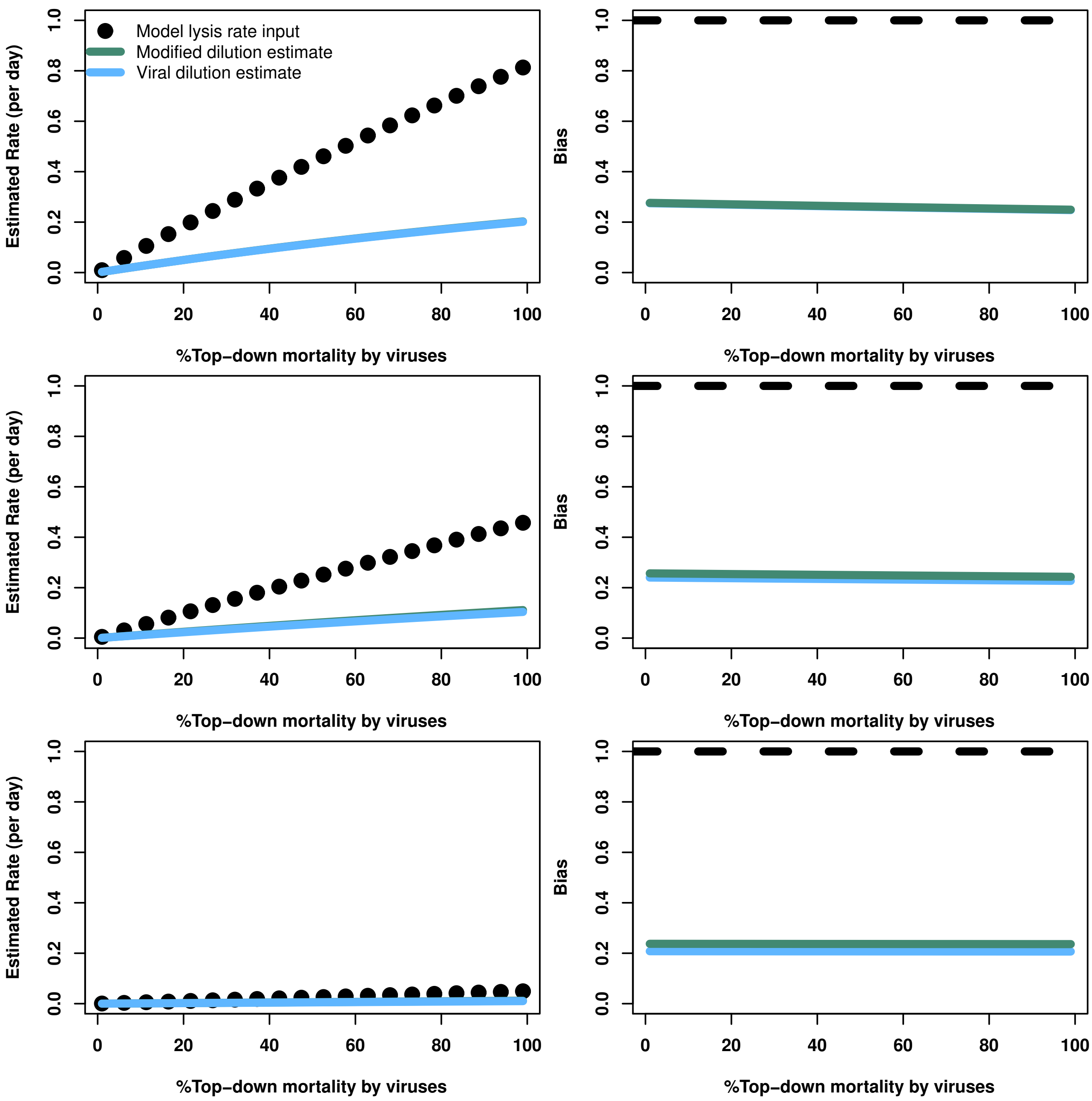

Figure S2. Rate estimates (left) and bias (right) of viral lysis rate in the infected class model with a 4 hour latent period calculated using a 2h incubation. Each row shows a different level of niche competition as indicated by the lines across Figure 2 (top: 5%, middle:50%, bottom: 95% niche competition).

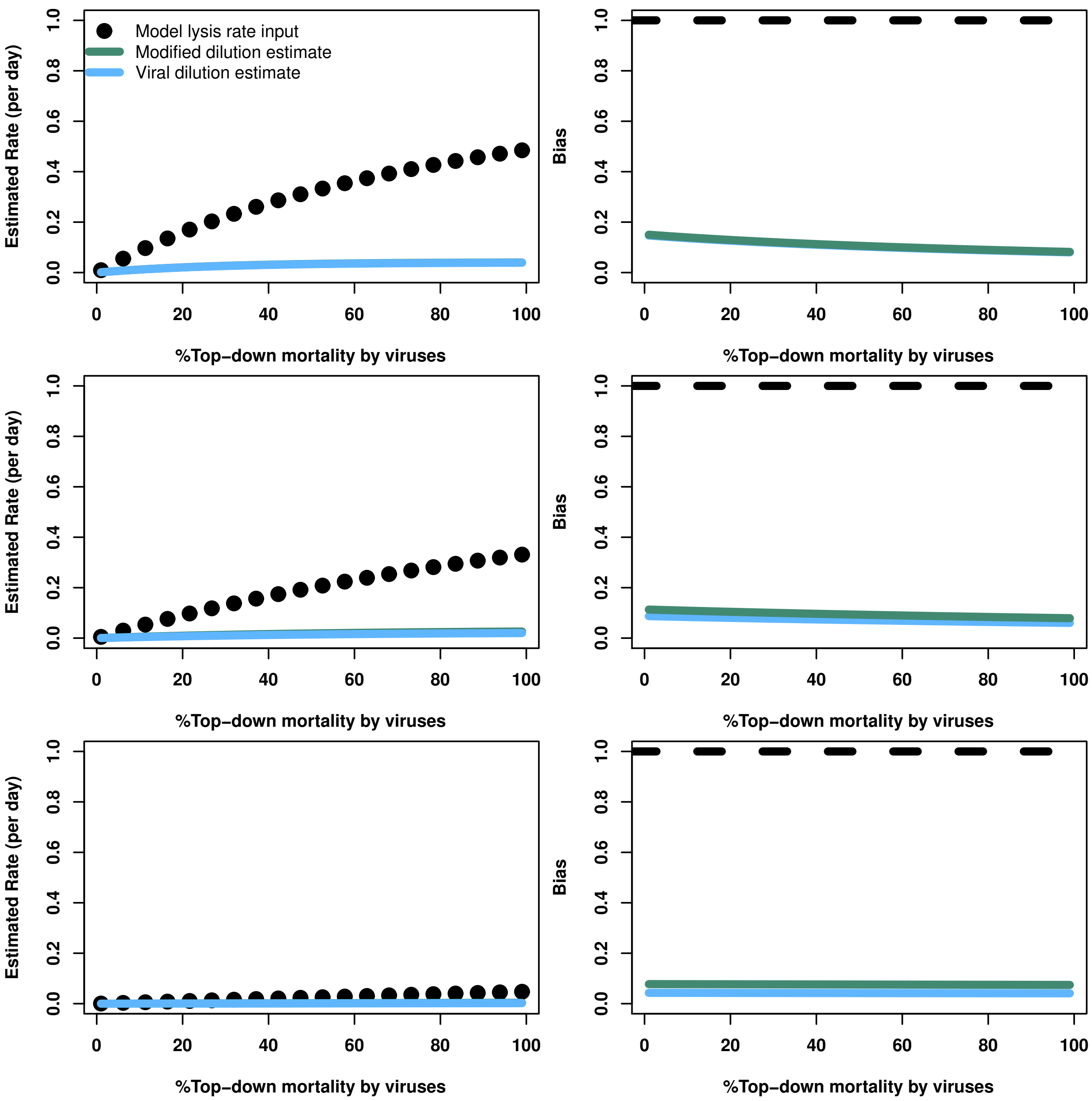

Figure S3. Rate estimates (left) and bias (right) of viral lysis rate in the infected class model with a 24 hour latent period calculated using a 2h incubation. Each row shows a different level of niche competition as indicated by the lines across Figure 2 (top: 5%, middle:50%, bottom: 95% niche competition).

SUPPLEMENTAL DATA F: INFECTED CLASS MODEL BEHAVIOUR 24H

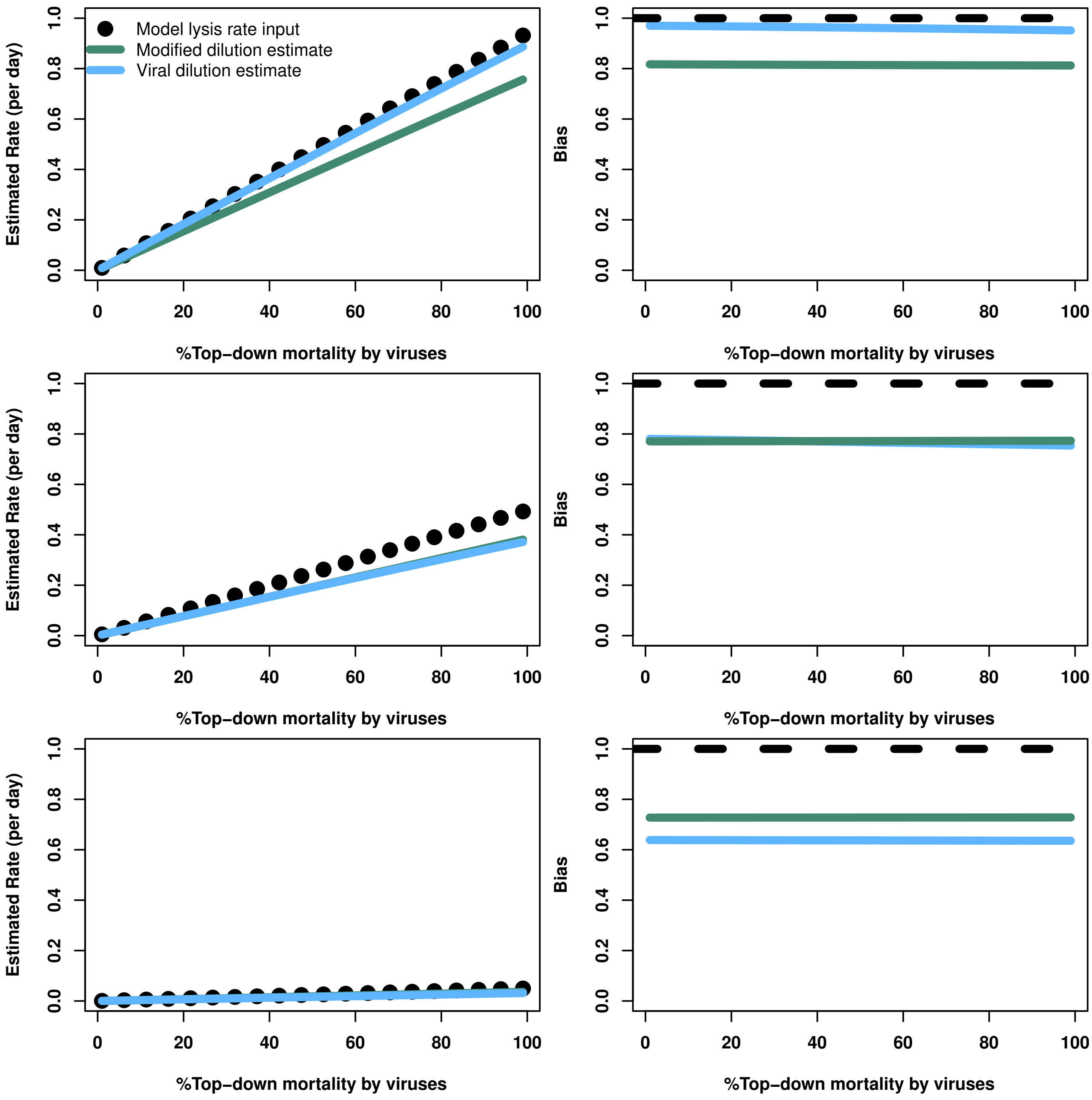

Figure S4. Rate estimates (left) and bias (right) of viral lysis rate in the infected class model with a 15 minute latent period calculated using a 24h incubation. Each row shows a different level of niche competition as indicated by the lines across 2 (top: 5%, middle:50%, bottom: 95% niche competition).

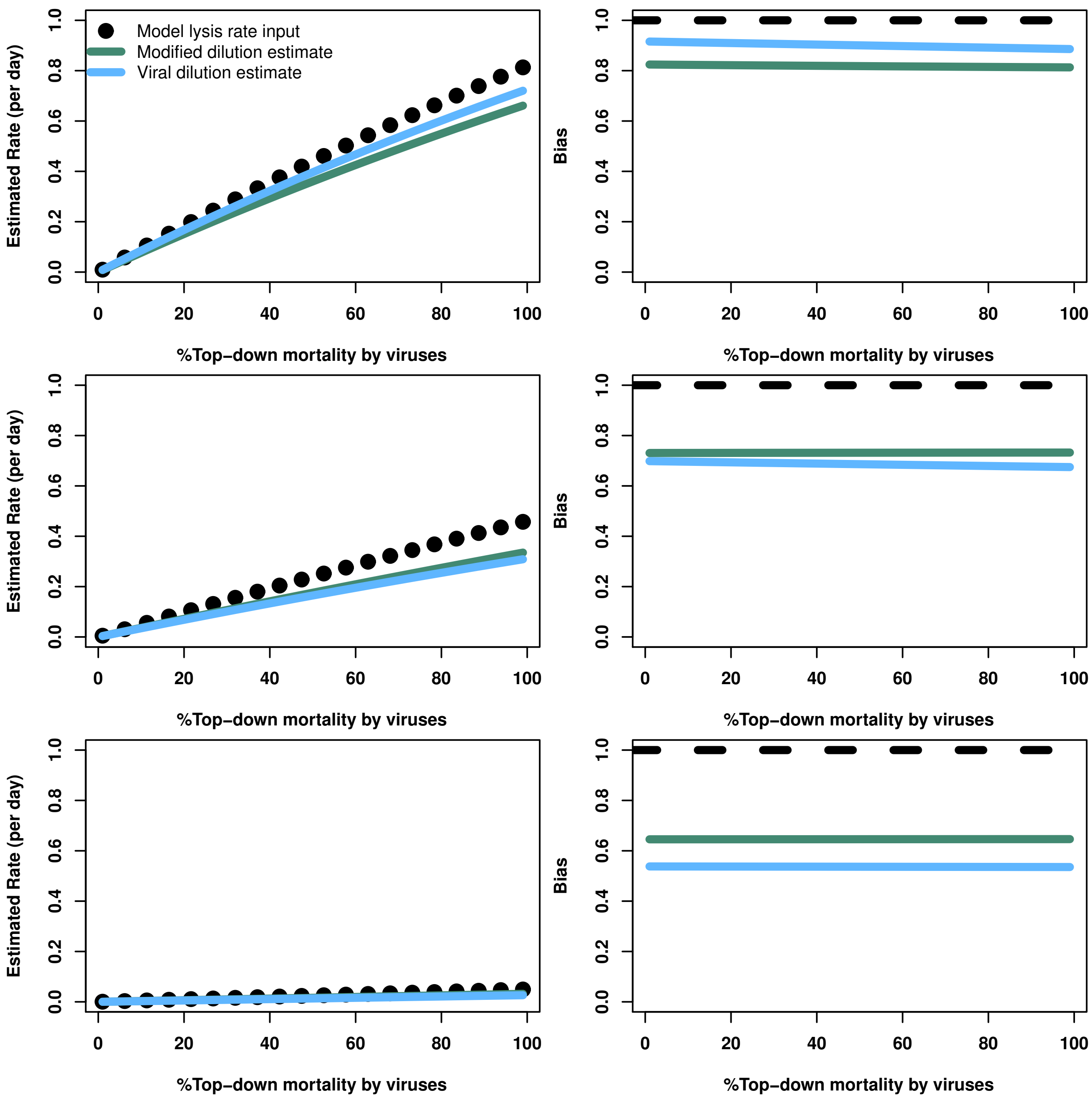

Figure S5. Rate estimates (left) and bias (right) of viral lysis rate in the infected class model with a 4 hour latent period calculated using a 24h incubation. Each row shows a different level of niche competition as indicated by the lines across Figure 2 (top: 5%, middle:50%, bottom: 95% niche competition).

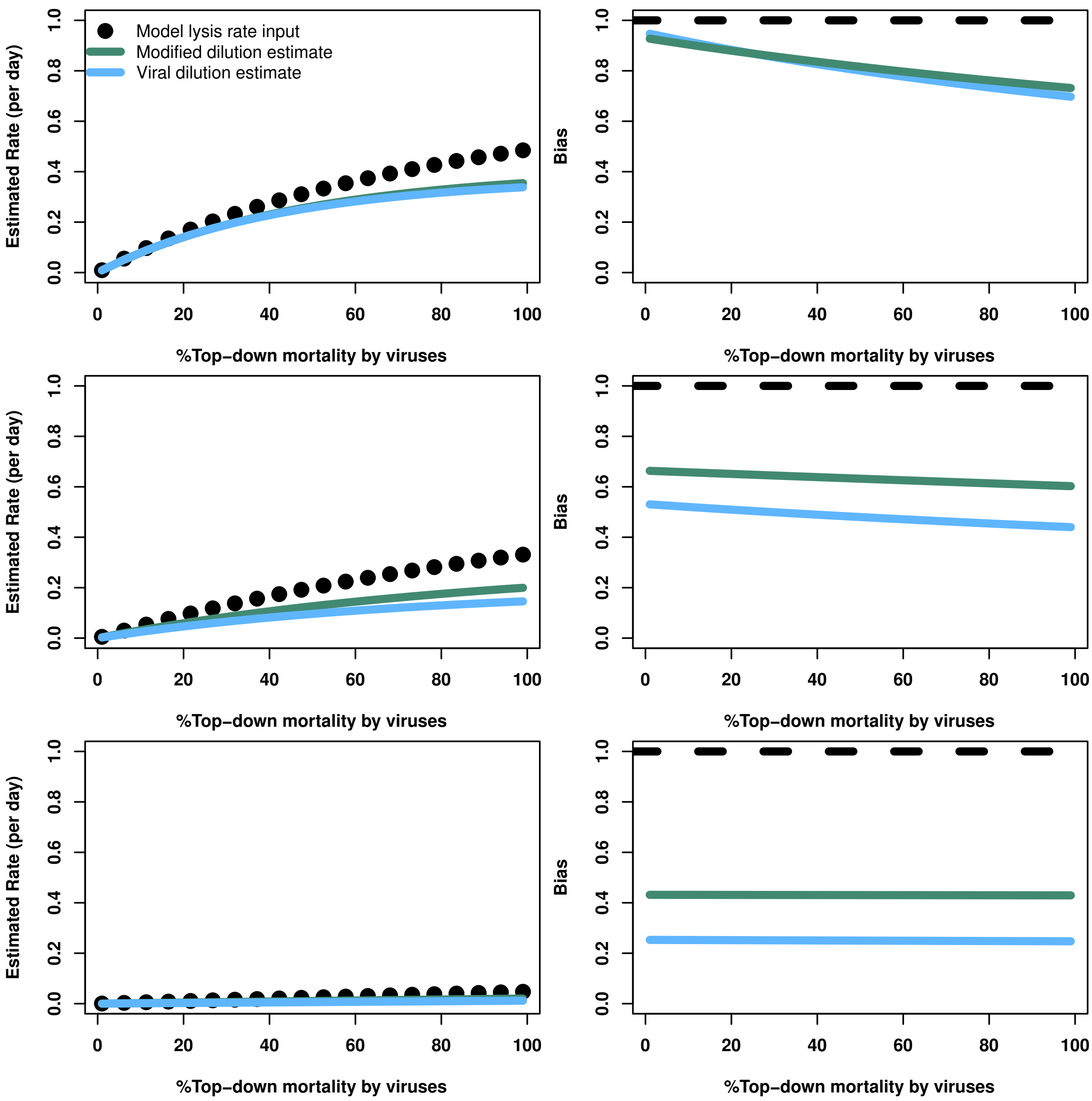

Figure S6. Rate estimates (left) and bias (right) of viral lysis rate in the infected class model with a 24 hour latent period calculated using a 24h incubation. Each row shows a different level of niche competition as indicated by the lines across Figure 2 (top: 5%, middle:50%, bottom: 95% niche competition).
